# Structural Features of a Full-Length Ubiquitin Ligase Responsible for the Formation of Patches at the Plasma Membrane

**DOI:** 10.3390/ijms22179455

**Published:** 2021-08-31

**Authors:** Jan Knop, Tim Lienemann, Haifa El-Kilani, Sven Falke, Catharina Krings, Maria Sindalovskaya, Johannes Bergler, Christian Betzel, Stefan Hoth

**Affiliations:** 1 Molecular Plant Physiology, Institute of Plant Science and Microbiology, Universität Hamburg, 22609 Hamburg, Germany; jan.knop@uni-hamburg.de (J.K.); tim.lienemann@uni-hamburg.de (T.L.); catharina.brieske@uni-hamburg.de (C.K.); maria.sindalovskaya@studium.uni-hamburg.de (M.S.); johannes.bergler@uni-hamburg.de (J.B.); 2 Institute of Biochemistry and Molecular Biology, Universität Hamburg, 20146 Hamburg, Germany; elkilani@biochem.uni-luebeck.de (H.E.-K.); falke@chemie.uni-hamburg.de (S.F.); Christian.Betzel@uni-hamburg.de (C.B.); 3 Laboratory for Structural Biology of Infection and Inflammation, c/o DESY, 22607 Hamburg, Germany

**Keywords:** SAUL1, ubiquitin ligase, U-box, armadillo repeats, multi-vesicular bodies, vesicle transport

## Abstract

Plant U-box armadillo repeat (PUB-ARM) ubiquitin (Ub) ligases have important functions in plant defense through the ubiquitination of target proteins. Defense against pathogens involves vesicle trafficking and the formation of extracellular vesicles. The PUB-ARM protein SENESCENCE ASSOCIATED UBIQUITIN E3 LIGASE1 (SAUL1) can form patches at the plasma membrane related to tethering multi-vesicular bodies (MVBs) to the plasma membrane. We uncovered the structure of a full-length plant ubiquitin ligase and the structural requirements of SAUL1, which are crucial for its function in patch formation. We resolved the structure of SAUL1 monomers by small-angle X-ray scattering (SAXS). The SAUL1 model showed that SAUL1 consists of two domains: a domain containing the N-terminal U-box and armadillo (ARM) repeats and the C-terminal ARM repeat domain, which includes a positively charged groove. We showed that all C-terminal ARM repeats are essential for patch formation and that this function requires arginine residue at position 736. By applying SAXS to polydisperse SAUL1 systems, the oligomerization of SAUL1 is detectable, with SAUL1 tetramers being the most prominent oligomers at higher concentrations. The oligomerization domain consists of the N-terminal U-box and some N-terminal ARM repeats. Deleting the U-box resulted in the promotion of the SAUL1 tethering function. Our findings indicate that structural changes in SAUL1 may be fundamental to its function in forming patches at the plasma membrane.

## 1. Introduction

Plants are constantly challenged by pathogen infections. However, they evolved specific and sensitive mechanisms for recognizing pathogens to initiate defense responses and can often successfully resist pathogen attacks. Two types of immune receptors can activate plant defense responses [1]. At the plasma membrane, pattern recognition receptors (PRR) perceive pathogen-associated molecular patterns (PAMP), such as bacterial flagellin and fungal chitin, to induce PAMP-triggered immunity (PTI), leading to efficient pathogen defense [2,3]. However, many pathogens have evolved systems that secrete effectors into eukaryotic host cells, aiming to inhibit immune signaling at the level of receptors or downstream signaling components [4,5]. To prevent these effectors from causing disease, plant cells express the second type of immune receptors, nucleotide-binding leucine-rich repeat proteins, which monitor the presence or activity of pathogen effectors in the cytosol to initiate effector-triggered immunity (ETI) [6]. The activation of nucleotide-binding leucine-rich repeat proteins results in a defense program similar to PRR-induced defense mechanisms, but ETI, in contrast to PTI, ultimately leads to cell death.

One of the major post-translational regulatory mechanisms of plant defense response is the ubiquitination of immune signaling components through E3 ubiquitin (Ub) ligases. E3 Ub ligases mediate specificity in ubiquitination by binding to the target substrate. They pair with Ub-conjugating enzymes E2 to form a ubiquitination complex that receives Ub from the Ub-activating enzyme E1 and transfers it to target proteins. The type of Ub chain transferred to the target determines whether the substrate protein is modulated by its activity or degraded by the 26S proteasome. Besides Cullin-RING-like multi-component complexes and RING ubiquitin ligases, plant U-box armadillo repeat (PUB-ARM) Ub ligases, representing a subgroup of the PUB protein family, are important for the regulation of plant defense [7,8,9]. Whereas the U-box of PUB-ARM proteins facilitates binding to the E2 enzyme [10,11], ARM repeats often consist of three α-helices, and domains of several ARM repeats form interfaces for protein-protein interactions and possibly for target recognition [12,13].

The Arabidopsis PUB-ARM proteins AtPUB12 and AtPUB13 recognize the PRR FLAGELLIN SENSING2 and mediate its poly-ubiquitination, thus leading to flagellin-induced degradation of FLAGELLIN SENSING2. Accordingly, *pub12* and *pub13* mutants showed increased immunity upon flagellin treatment [14]. Recently, AtPUB13 was found to be involved in the regulation of another PRR, the chitin receptor LYSIN MOTIF RECEPTOR KINASE5 (LYK5), indicating that PUB-ARM Ub ligases may have multiple substrates in vivo [15]. This finding is further supported by the degradation of the protein phosphatase ABA INSENSITIVE1 (ABI1), which requires AtPUB12 and AtPUB13 [16]. The rice ortholog of AtPUB13 and SPOTTED LEAF11 (SPL11) also negatively regulates pathogen resistance [17]. The protein kinase BIK1 that phosphorylates FLAGELLIN SENSING2 is targeted for proteasomal degradation by the PUB-ARM proteins AtPUB25 and AtPUB26. Compared to wild-type plants, *pub25*, *pub26*, and *pub25 pub26* mutants showed decreased pathogen susceptibility, thus supporting the roles of AtPUB25 and AtPUB26 in Arabidopsis immunity [18]. A triplet of E3 ubiquitin ligases, consisting of AtPUB23, AtPUB24, and AtPUB25, mediated negative regulation of PTI in Arabidopsis [19]. Consequently, AtPUB22 has been shown to mediate ubiquitination and thus degrade the exocyst subunit EXO70B2 to regulate PTI [20]. Several PUB-ARM proteins achieve positive regulation of immunity, including Avr/Cf-9 RAPIDLY ELICITED74 (ACRE74, also named NtCMPG1) and ACRE276 from tobacco. Whereas downregulation of *NtCMPG1* resulted in reduced immune responses, its overexpression induced stronger immunity [21]. The loss of *ACRE276* also led to diminished defense responses, which may be rescued by expressing its Arabidopsis ortholog AtPUB17, thus proposing that both act as positive regulators of immunity [22]. Positive regulation of immune responses was also suggested for *OsPUB15* and *OsPUB44* from rice and *MdPUB29* from apples [23,24,25].

Previously, we identified the PUB-ARM protein SENESCENCE-ASSOCIATED E3 UBIQUITIN LIGASE1 (SAUL1) as a positive regulator of PTI [26]. The homeostasis of SAUL1 appears to be strongly guarded. The absence of SAUL1 in *saul1* mutants, which leads to temperature-dependent autoimmune responses in all above-ground organs [27,28], is guarded by a complex between the TIR-NB-LRR protein suppressors *chs1-2*, 3 (SOC3) and the TIR-NB protein CHILLING SENSITIVE1 (CHS1) [26]. By contrast, the overexpression of SAUL1, which results in reduced growth and increased resistance [26,29], is guarded by a complex between the TIR-NB-LRR protein suppressors *chs1-2*, 3 (SOC3) and the TIR-NB protein TIR-NBS2 (TN2) [30]. SAUL1-type PUB-ARM proteins contain an elongated C-terminus compared with other PUB-ARM proteins and are localized to the plasma membrane (PM) through ARM repeats in their elongated C-terminus. This additional ARM repeat domain is essential and sufficient for targeting SAUL1 to the PM, though this domain by itself is localized to large patches in the PM [29,31]. A SAUL1 target at the PM is not yet known. The localization of the membrane patches allowed us to suggest a function for SAUL1 in tethering multi-vesicular bodies (MVBs) and/or the tonoplast (TP) to the PM. The size of such patches is generally dependent on the expression level [32]. Both full-length GFP-SAUL1 or YFP-SAUL1 fusion proteins, as well as fusions between GFP and the C-terminal ARM repeat domain of SAUL1, could mediate patch formation [31,32]. Interestingly, patch formation and consequently tethering were induced by the infection of *35S::GFP-SAUL1* plants with the oomycete *Phytophthora capsici* [32]. However, unlike GFP-SAUL1, full-length SAUL1-GFP fusion proteins were not localized to membrane patches, suggesting that the SAUL1 C-terminus may need to remain unmodified to mediate patch formation [31].

It can hardly be predicted from available structural data how the overall structure of the U-box and the ARM repeats of SAUL1 relate to its functions since the structure of a plant’s full-length ubiquitin ligase has not been published yet. The structure of the U-box domain of the SAUL1 Arabidopsis paralog AtPUB14 was resolved by NMR [33]. Generally, the U-box is structurally similar to RING-finger domains lacking metal-binding residues and contains a hydrophobic groove for potential interaction with E2 enzymes, mediating dimerization [33,34,35,36]. The structure of an ARM repeat domain, which forms a superhelix of helices for potential interaction with other molecules, was first resolved for murine β-catenin [12]. The yeast ubiquitin ligase Ufd2p, like SAUL1, consists of multiple ARM-like repeats, and a U-box with an elongated shape derived from its crystal structure [36]. In this study, we aimed to unravel the structure of a full-length plant ubiquitin ligase and the structural requirements in SAUL1 that may allow for patch formation at the PM.

## 2. Results

### 2.1. SAUL1 Domain for the Formation of Patches at the PM

**Figure 1:**
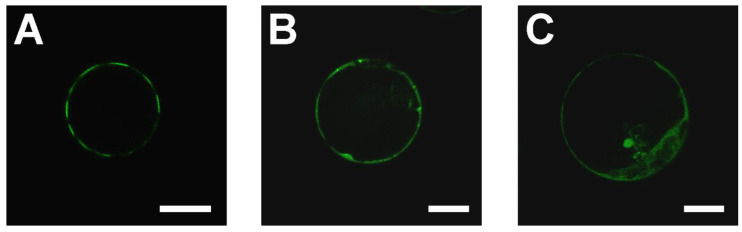
Analysis of the SAUL1 domain responsible for its function in patch formation. (**A**) GFP fluorescence of *GFP-SAUL1ARM*_7–11_ following expression in mesophyll protoplasts, indicating patch formation. Patch formation was lost following the deletion of ARM repeats in *GFP-SAUL1ARM*_8–11_ (**B**) and *GFP-SAUL1ARM*_7–10_ (**C**). Scale bars represent 15 µm.

The SAUL1 ubiquitin ligase can be found in patches at the PM, and the C-terminal ARM_7–11_ domain of SAUL1 is sufficient to form these patches and possibly tether multi-vesicular bodies (MVBs) and/or the tonoplast (TP) to the PM [31,32]. In an attempt to identify potential binding sites in SAUL1-ARM_7–11_ for the PM or MVBs/TP, we created GFP fusion proteins with only four ARM repeats and studied their fluorescence following expression in Arabidopsis leaf cell protoplast by confocal laser scanning microscopy. Whereas fluorescence signals indicated patch formation in all 100 of the *35S::GFP-SAUL1ARM*_7–11_ protoplasts analyzed (Figure 1A), the removal of either ARM repeat 7 or 11 resulted in the loss of PM association and patch formation in 100% of the *35S::GFP-ARM*_8–11_ and *35S::GFP-ARM*_7–10_ protoplasts, respectively. Both fusion proteins GFP-ARM_8–11_ and GFP-ARM_7–10_ showed fluorescence signals in the cytosol (Figure 1B,C, *n* = 100 protoplasts), suggesting that an intact ARM repeat domain is required for the formation of patches at the PM.

### 2.2. Structure of SAUL1 Monomers

To investigate the structural requirements of the SAUL1 protein for binding to the PM and potentially MVBs/TP, we used the pGEX-6p-1 vector to recombinantly express N-terminally GST-tagged SAUL1 in *E. coli*. SDS-PAGE analysis revealed a distinct protein band at the expected size of GST-SAUL1 at 115.2 kDa (Supplementary Figure S1A). Mass spectrometry confirmed the identity of GST-SAUL1 in that band. In the next step, affinity chromatography was performed and led to identifying an absorbance peak resembling GST-SAUL1 (Supplementary Figure S1B). The respective fractions were used to proteolytically cleave the GST-tag from SAUL1. We used size exclusion chromatography (SEC) to successfully separate the proteins SAUL1 and GST from each other (Supplementary Figure S1C,D). We applied circular dichroism spectroscopy to confirm that the purified SAUL1 proteins were folded in a native manner. This analysis indicated that SAUL1 comprised 66.3% α-helices, which aligned with the presence of 11 ARM repeats in the protein, 15.4% β-sheets, 6.4% turns, and 11.9% random regions (Supplementary Figure S2), indicating that SAUL1 was structurally ordered. The native SAUL1 was therefore used for structural analysis in solution by small-angle X-ray scattering (SAXS).

**Figure 2:**
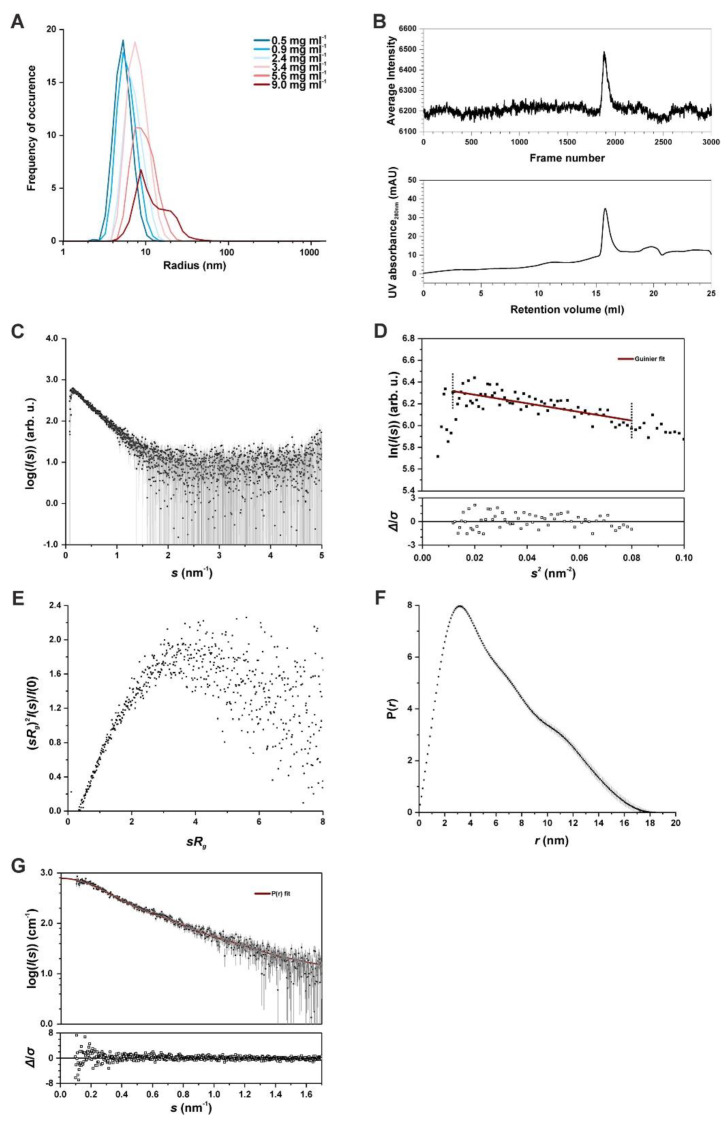
Structural analysis of SAUL1 monomers. (**A**) DLS measurements of different SAUL1 concentrations ranging from 0.5 to 9.0 mg mL^−1^ in 50 mM Tris-HCl pH 9 and 250 mM NaCl. Detected radii shift to higher values and multiple radii with a protein concentration higher than 2.4 mg mL^−1^. (**B**) Scattering intensities of monodisperse SAUL1 in the SEC-SAXS experiment with a loading concentration of 4.38 mg mL^−1^ and chromatogram of the UV absorption at 280 nm. (**C**) Scattering data of SAUL1. Log-linear plot of I(*s*) versus *s*. (**D**) The upper plot shows the Guinier fit (red) of the SAXS data. Dotted lines mark the fit range (*s*_min_ = 0.005 nm^−1^ and *sR_g_* max = 1.3). The lower plot shows the standardized residual plot. (**E**) Dimensionless Kratky plot with the intensities normalized to the forward scattering intensity (*I*(0)) and the radius of gyration (*R_g_*). (**F**) P(*r*) versus *r* profile of SAUL1. (**G**) Fit of the P(*r*) function (red) to the experimental SAXS data. The lower plot depicts a standardized residual plot.

To test for the monodispersity of the SAUL1 solution in preparation for SAXS, dynamic light scattering (DLS) was used to detect the particle radii present in the respective solution and define the hydrodynamic radius distribution of SAUL1. The tested concentrations of SAUL1 solutions ranged from 0.5 to 9.0 mg mL^−1^. A monodisperse SAUL1 solution with a hydrodynamic radius below 6 nm was observed with concentrations less than or equal to 2.4 mg mL^−1^ (5.2 ± 0.1 nm at 0.5 mg mL^−1^; 5.7 ± 0.4 nm at 0.9 mg mL^−1^). By contrast, the hydrodynamic radius increased to 7.0 ± 0.8 nm and 9.1 ± 1.7 nm at concentrations higher than 2.4 mg mL^−1^ or above 5 mg mL^−1^, respectively. In the latter range, the radius distribution was also much broader (Figure 2A).

The SAUL1 solution consecutively used for inline SEC-SAXS had an initial concentration of 4.4 mg mL^−1^ to warrant that the concentration was in the range of monodispersity but not too low in the actual SAXS measurement. During the inline SEC, which directly connects to the SAXS sample exposure unit [37], one absorbance peak with an intensity of about 30 mAU was detected (Figure 2B). The log-linear plot of the scattering data showed a smooth decrease toward higher angles, indicating that SAUL1 may contain unfolded regions (Figure 2C). The non-sigmoidal shape of the plot may suggest that SAUL1 did not have a globular, but an elongated shape. The radius of gyration R_g_ was determined at 4.64 ± 0.54 nm based on the Guinier approximation (Figure 2D). The dimensionless Kratky plot was applied to assess the overall shape of SAUL1. The intensities increased to a maximum of 3.2 and decreased at higher angles with a lower slope (Figure 2E). Nevertheless, the curve may be considered bell-shaped but right-shifted, again pointing to the elongated and locally flexible shape of SAUL1. A non-Gaussian-shaped curve with a peak of approximately 3.15 nm was obtained by calculating the P(*r*) function, which verified the significantly elongated shape of SAUL1 (Figure 2F,G). The maximum diameter *d*_max_ was determined at 18.4 nm as the x-axis intercept coordinate of the P(*r*) function. The underlying fit to the experimental data that verify the calculation of the P(*r*) function via Fourier transform had an estimated quality of 0.77; most standardized residuals were within the range of two, correlating with a rod-shaped elongated protein.

**Figure 3:**
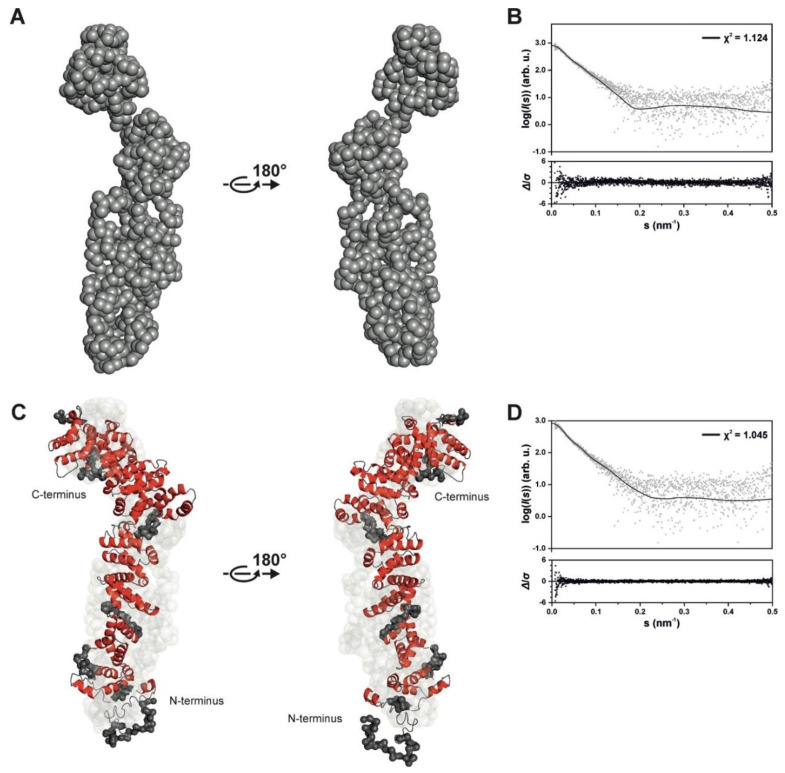
Ab initio and in silico structures of SAUL1 derived from SEC-SAXS measurements. (**A**) Ab initio bead model of SAUL1 with a resolution of 45 ± 3 Å and normalized spatial discrepancy of 1.498. (**B**) Fit of the ab initio model to the raw scattering data with the standardized residual plot Δ/*σ* = (*I*_exp_(*s*) − *I*_mod_(*s*))/*σ*(*s*). (**C**) *I-TASSER* derived in silico model, subdivided into different domains upon an *InterPro* analysis and refined using *EOM*. Depicted are α-helices (red), β-sheets (blue), loops (gray), and flexible regions (gray). (**D**) Fit of the in silico model to the raw scattering data with the standardized residual plot.

In the next step, we generated an ab initio model of SAUL1 by using *GASBOR*. The most likely model had a resolution of 45 ± 3 Å and a normalized spatial discrepancy value of 1.498 (Figure 3A). To compare this model with the original scattering data, a theoretical scattering curve of this structure was calculated using *CRYSOL*. This comparison resulted in a low χ^2^ value of 1.124, thus demonstrating that the ab initio model represented the experimental data effectively (Figure 3B).

According to all previous calculations, SAUL1 was present in an elongated rod-like structure; the rod had a wide, even longitudinal axis and was slightly bent at one end. Subsequently, we applied in silico modeling for further elucidation of the structural organization of SAUL1. A sub-divided *I-TASSER* model based on the prediction of rigid regions in SAUL1 was used for calculations with the ensemble optimization method (EOM). The resulting in silico model contained five different states, with model 1 being the most abundant state (Supplementary Figure S3A–C). Quality assessment by *CRYSOL* indicated that the models fit effectively to the experimental data (Figure 3C,D). At the N-terminus, which contains the U-box, the overall shape of the model was slightly expanded. However, most regions were quite condensed, and most predicted domains appeared to be folded correctly.

**Figure 4:**
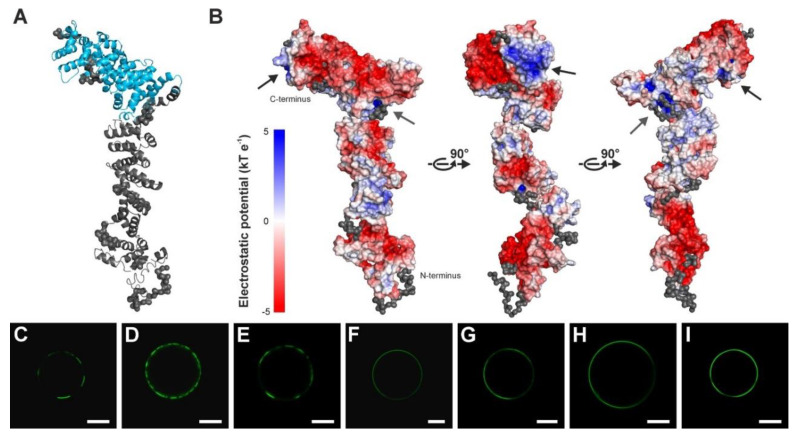
Structural model of the SAUL1 monomer. (**A**) *EOM* model of SAUL1. Potential ARM repeats 7–11, which are responsible for the localization of SAUL1 at the plasma membrane, are marked in blue. (**B**) Electrostatic potential of the solvent-accessible surface of SAUL1 in a range of ± 5 kT e^−1^. Calculations were performed by splitting the obtained SAUL1 model into two regions: one containing the potential ARM repeats 7–11 and the rest of the protein in the other. Blue and red areas represent positively charged and negatively charged regions, respectively. The positively charged groove next to the C-terminus in the area of the potential ARM repeats 7–11, consisting of Arg736, Arg737, and Arg775, which is marked by a black arrow. (**C**–**I**) GFP signals of arginine mutants. GFP fluorescence in protoplasts following protein expression of the SAUL1 R775A single mutant (**C**), the SAUL1 R737A single mutant (**D**), the SAUL1 R737A/R775A double mutant (**E**), the SAUL1 R736A single mutant (**F**), the SAUL1 R736A/R775A double mutant (**G**), the SAUL1 R736A/R737A double mutant (**H**), and the R336A/R737A/R775A triple mutant (**I**). Scale bars in (**C**–**I**) represent 15 µm.

The SAUL1 model indicated that SAUL1 might consist of two major parts: one containing the N-terminal U-box and the ARM repeats 1 to 6, and the other containing the C-terminal ARM repeats 7 to 11 (Figure 4A). The C-terminus of SAUL1 has previously been shown to mediate plasma membrane association [31]. In addition, this domain is important for PM interactions with the tonoplast or multi-vesicular bodies [32]. These interactions may involve the binding of positively charged regions in a protein to membrane lipids. To test whether SAUL1 has this property, the electrostatic surface potential was determined by splitting the model of SAUL1 into two domains and analyzing the structurally ordered regions. A distinct positively charged groove at the C-terminal membrane association domain containing arginine residues R736, R737, and R775 was detected (Figure 4B). In addition, two large negatively charged regions were discovered. Whereas one was localized at the N-terminus close to the U-box, the other was found in the C-terminal ARM repeat region opposite the positively charged groove (Figure 4B). In an attempt to investigate the contribution of the positively charged groove to the function of SAUL1 in patch formation, we generated point mutations in the respective arginine residues of the full-length SAUL1 protein and studied patch formation in Arabidopsis protoplasts. The unchanged full-length GFP-SAUL1 protein mediated patch formation in 60.5% of the protoplasts analyzed (n = 4228 protoplasts). In line with the fact that R775 was not contained in the ARM7-11 construct, which was sufficient to form patches (c.f. Figure 1A), the SAUL1 R775A single mutant formed patches in a comparable proportion to protoplasts (69.0%, n = 3194 protoplasts) transformed with *35S::GFP-SAUL1 R775A* (Figure 4C). Whereas SAUL1 R737A single mutants formed patches in 53.2% of protoplasts (n = 3514 protoplasts), the proportion of protoplasts exhibiting patch formation was slightly reduced in SAUL1 R737A/R775A double mutants (36.8%, n = 3912 protoplasts) (Figure 4D,E). The mutation of arginine at position 736 in SAUL1 nearly resulted in the complete loss of patch formation. The proportion of protoplasts forming patches was reduced to 2.9% in SAUL1 R736A single mutants (n = 3054 protoplasts), 2.9% in SAUL1 R736A/R775A double mutants (n = 3669 protoplasts), 3.2% in SAUL1 R736A/R737A double mutants (n = 3624 protoplasts), and 1.4% in R336A/R737A/R775A triple mutants (n = 3659 protoplasts) (Figure 4F–I), thus indicating that the arginine residue at position 736 has a crucial function in the tethering function of SAUL1. To exclude the possibility that the proposed function of this particular arginine residue is due to the loss of the mutant protein’s structure, we expressed and purified the SAUL1 R736A protein. The determined CD spectrum of SAUL1 R736A was identical to the CD spectrum of the SAUL1 protein (Supplementary Figure S2), indicating that the single amino acid change did not result in the loss of protein structure. We also co-localized the SAUL1 R736A/R737A/R775A GFP fusion protein with the fluorescent dye FM4-64, which stains the plasma membrane, to show that these arginine mutants are still localized to the plasma membrane (Supplementary Figure S4).

### 2.3. Structural Analysis of SAUL1 as a Polydisperse System

Determining the overall structure of SAUL1 monomers was a prerequisite to enable modeling of polydisperse and oligomeric systems. Therefore, we measured SAUL1 solutions with concentrations ranging from 0.43 to 4.96 mg mL^−1^ in regular SAXS batch measurements. The SAXS scattering data and the *I*(0) values as determined by the Guinier plot revealed concentration-dependent differences (Supplementary Figure S5A,B). The *I*(0) values increased at higher concentrations, indicating that larger particles were detected in those solutions. The calculated *Rg* values also increased toward higher concentrations, from 6.27 ± 3.00 nm up to 10.64 ± 1.55 nm, in an approximately linear manner (Supplementary Figure S5C). These results suggested that the polydisperse system contained multiple SAUL1 oligomers.

**Figure 5:**
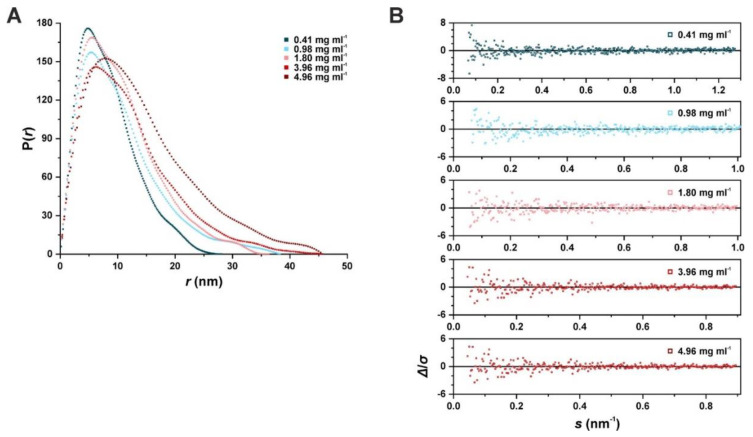
Radius distributions of SAUL1 in the batch measurement. (**A**) P(*r*) versus *r* profile of SAUL1 at different concentrations. (**B**) Plot of the different standardized residuals of the fits of the P(*r*) functions against the scattering data.

We used a dimensionless Kratky plot to compare the different average shapes of the putative SAUL1 oligomeric states. For all concentrations, the plot showed a non-bell-shaped curve that reached a plateau around 3. With increasing concentrations, the curves declined more gradually (Supplementary Figure S5D). The overall ab initio shape suggested that SAUL1 is present as an elongated and partially flexible protein. The *P*(*r*) distributions showed a semi-bell-shaped left-shifted curve with a maximum increasing toward higher concentrations (Figure 5A). The *d_max_* increased from 28.1 nm at a concentration of 0.43 mg mL^−1^ to 45.7 nm at 4.96 mg mL^−1^. All distributions represented the experimental data well, as the standardized residuals of the fit against the raw data were mostly below 2 and had a quality estimate value of approximately 70% (Figure 5B). The calculated molecular weight ranged from 177 kDa to 265 kDa, indicating that the composition of SAUL1 oligomers changed dynamically in those solutions.

**Figure 6:**
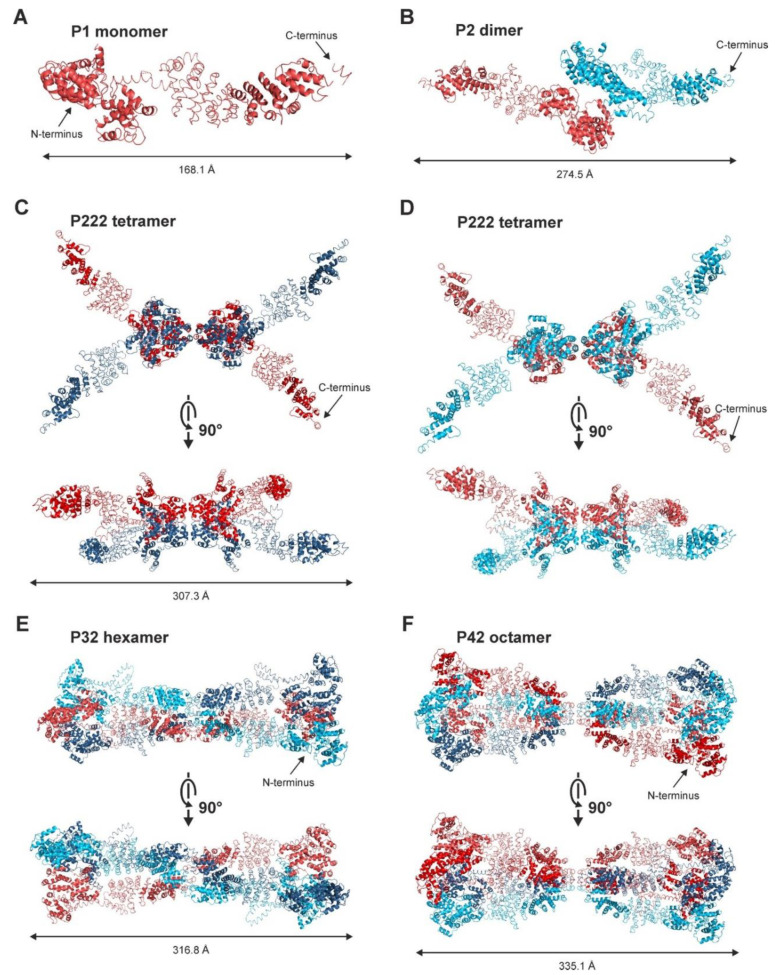
Oligomeric states of SAUL1. *SASREF* derived oligomeric models of SAUL1. Monomeric subunits are represented by one color. (**A**) The monomeric model was generated using SEC-SAXS experimental data. The model was built using *CORAL* and refined with *SREFLEX*. All oligomeric states are based on this model. (**B**) P2 dimer model fitted to a concentration of 0.43 mg ml^−1^. P222 tetrameric model fitted to SAUL1 solutions with concentrations of (**C**) 0.98 and (**D**) 3.96 mg mL^−1^. (**E**) The P32 hexameric and (**F**) P42 octameric models were fitted to a SAUL1 concentration of 0.43 mg mL^−1^.

Defining a suitable monomeric structure was essential to determine the fraction of different SAUL1 oligomers in the sample solutions. Since the SAUL1 protein was quite flexible, a monomeric model was generated based on ten domains, defined following the *I-TASSER* homology model and fitted to the SEC-SAXS scattering amplitudes using *SASREF*. In a second step, the obtained model was refined using *SREFLEX,* allowing for a more probable monomeric structure with a χ^2^ value of 1.084. This model was used to generate different multimeric structures using *SASREF* based on the data of the batch SAXS experiment. In the next step, combinations of multimers were used to calculate diverse mixtures by fitting them against the experimental data using *OLIGOMER*. The most likely oligomeric states in the different SAUL1 solutions were the SEC-SAXS-based P1 monomer, a P2 dimer, a P222 tetrameric structure, and a low fraction P32 hexameric and P42 octameric structures (Figure 6A–F). Accordingly, the putative oligomerization domain comprised the U-box, and parts of the armadillo (ARM) repeats 1 to 6. Whereas the dimer was the predominant structure at low SAUL1 concentrations, the tetrameric structure became predominant at higher concentrations (Supplementary Figure S6).

### 2.4. Relocalization of SAUL1 upon Masking or Deleting Its N-Terminus

**Figure 7:**
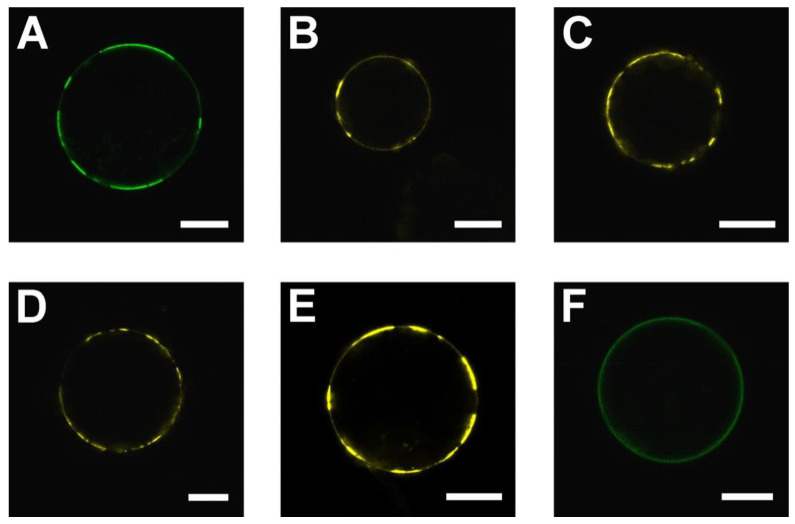
Impact of the SAUL1 N-terminus on its tethering function. (**A**) GFP fluorescence of SAUL1∆N-GFP expressed in mesophyll protoplasts. (**B**–**E**) BiFC analysis of the interplay between SAUL1 and different E2s. Mesophyll protoplasts transformed with BiFC constructs containing SAUL1 paired with *UBC10* (**B**), *UBC28* (**C**), *UBC29* (**D**), and *UBC30* (**E**). SAUL1 was always tagged C-terminally, whereas different UBCs were tagged at the N-terminus. (**F**) GFP signal of U-box-GFP-ARM_7-11_ following the expression in mesophyll protoplast. Scale bars represent 15 µm.

The SAUL1 C-terminus containing ARM repeats 7 to 11 was sufficient for patch formation and, thus, potential PM-MVB/TP tethering (c.f. Figure 1A), thus proposing that tethering does not require the tetramerization of SAUL1 via the N-terminus. In addition, patch formation was only observed in plant cells expressing GFP-SAUL1 but not in plant cells expressing SAUL1-GFP [31]; this may indicate that patch formation in the SAUL1 full-length protein may require some hindrance for oligomerization through the masking of the N-terminus by GFP. To test the hypothesis of whether masking or deleting the N-terminus allows for patch formation, we deleted 93 amino acids from the N-terminus to affect the U-box and ARM repeat domains 1 to 6 and potentially prevent oligomerization. We fused the GFP to the C-terminus of the deleted SAUL1∆N protein. The fluorescence signal of SAUL1∆N-GFP was observed in patches at the PM in all 100 protoplasts analyzed (Figure 7A), indicating that the disturbance to oligomerization helped allow for patch formation via the SAUL1 C-terminal domain.

Ubiquitin-conjugating (UBC)/E2 enzymes were previously shown to bind to the U-box of E3 ubiquitin ligases [10,11]. To test whether the binding of UBCs to the U-box of E3 ligases affects oligomerization, we studied patch formation through BiFC with SAUL1 and different UBCs. BiFC complexes containing SAUL1 and UBC10, UBC28, UBC29, or UBC30, showed YFP signals in patches at the PM of protoplasts analyzed (Figure 7B–E, n = 100 protoplasts for each UBC). Patch formation preferentially occurred upon masking or deleting the U-box; thus, oligomerization of the SAUL1 protein was prevented. We hypothesized that this model, which introduces the U-box N-terminal to the GFP-ARM_7–11_ fusion protein, may again suppress patch formation and possibly PM-MVB/TP tethering by oligomerization. When expressing U-box-GFP-ARM_7–11_ fusion proteins in protoplasts, the GFP signal was distributed uniformly at the plasma membrane in all 100 protoplasts analyzed, thus indicating that patch formation did not occur (Figure 7F).

## 3. Discussion

In this study, we resolved the structure of a full-length plant ubiquitin ligase, SAUL1, which belongs to the family of PUB-ARM proteins. These plant-specific proteins contain an N-terminal U-box followed by a variable number of armadillo (ARM) repeats [9,38]. Generally, a U-box facilitates the binding of E2 enzymes, presumably through hydrophobic residues [34]. ARM repeats generally consist of three α-helices, and several ARM repeats form surfaces that enable the binding of interacting proteins [12,13,39]. In addition, in SAUL1-type PUB-ARM proteins, the C-terminal ARM repeats are necessary and essential for association with the plasma membrane (PM) [29,31].

Applying SEC-SAXS to the recombinant protein showed that the SAUL1 monomer has an elongated structure (Figure 3 and Figure 4). The yeast ubiquitin ligase Ufd2p, consisting of multiple ARM-like repeats and a C-terminal U-box, demonstrated a similar elongated shape [36]. The elongated structure of SAUL1 was bent at one end due to the flexibility of the ARM repeats in the protein. This intrinsic flexibility of ARM repeats has also been described for the adenomatous polyposis coli protein and human β-catenin [12,40,41]. The derived structural model suggested that SAUL1 consists of mainly two parts: one part contains the U-box and the first few ARM repeats, and the other only consists of ARM repeats, particularly the C-terminal ARM repeats 7 to 11. Modeling the close SAUL1 paralog PUB43, which is also associated with the PM and potentially mediates the tethering of multi-vesicular bodies (MVBs) and/or the tonoplast (TP) to the PM [29,32], to the SAUL1 structural data, we showed a similar separation into two parts for PUB43 (Supplementary Figure S7).

This separation may be in good accordance with a dual function of SAUL1. The ARM 7–11 domain in the second part of SAUL1 has been shown to mediate association with the plasma membrane [29,31]. In addition, this domain was essential for the suggested tethering function of SAUL1, which connects MVBs and/or the TP to the PM, leading to the formation of patches [31,32]. It is not yet clear how SAUL1 binds to membrane compartments. ARM repeats mostly from important domains for protein–protein interactions, thus implying that other membrane proteins may be crucial for the association of SAUL1 with membranes. However, previous evidence suggests that PUB-ARM proteins interact with phosphoinositides through their ARM repeats [42]. Membranes containing such phosphoinositides are normally negatively charged. The binding of a protein to this membrane domain therefore requires a positively charged protein domain. In alignment with this, we identified a positively charged groove in the C-terminus of SAUL1 containing three arginine residues at positions 736, 737, and 775 (Figure 4). Two of these arginine residues were conserved in PUB43 (Arg747 and Arg785), and an additional arginine residue was found at position 745 in PUB43. Through site-directed mutagenesis and confocal analysis of the localization of single, double, and triple mutant proteins, we demonstrated that R736 was essential for the potential tethering function of SAUl1. The ability to form patches was lost in SAUL1 R736A, SAUL1 R736A/R775A single, SAUL1 R737A/R775A double, and R736A/R737A/R775A triple mutants, though the mutant proteins were still localized to the plasma membrane (Figure 4F–I, Supplementary Figure S4).

In the first part of the SAUL1 protein, the U-box may serve the binding of E2 enzymes for the SAUL1 ubiquitin ligase function [27,43]. The deletion of the U-box, or the mutation of the conserved C29 residue predicted as essential for ubiquitin ligase activity, prevented the complementation of the *saul1-1* mutant phenotype [26]. Our derived structure of the SAUL1 U-box resembled the resolved structure of other U-boxes [33,34,35,36]. The similarities between the U-box structure and other available structures suggests that the binding of E2 enzymes is also mediated by the U-box in the SAUL1 ubiquitin ligase.

Additionally, the U-box was involved in forming SAUL1 oligomers. We were able to model oligomeric SAUL1 structures from polydisperse systems based on the monomeric structure (Figure 6). Oligomer formation occurred via the U-box and parts of the first set of ARM repeats. It was previously published that U-boxes are fundamental to the dimerization of mouse CHIP and oligomerization of yeast Prp19 [34,35]. In plants, U-box-dependent dimerization has been shown for the PUB-ARM protein PUB22 in vivo [44]. The important role of ARM repeats in dimerization was observed for PUB10 during in vitro experiments [45]. For both PUB10 and PUB22, protein activity depended on their dimerization state. By contrast, PUB10 was only active as a homodimer and only monomeric PUB22 bound to its targets. PUB22 homo-dimerization led to auto-ubiquitination, keeping the abundance of PUB22 relatively low [44,45]. Several pieces of data supported the idea that oligomerization may also be important to patch formation through SAUL1. However, oligomerization via the U-box was not important for tethering MVBs and/or the TP to the PM or patch formation because fluorescent patches were prominent in *35S::GFP-SAUL1ARM*_7–11_ and *35S::SAUL1∆N-GFP* protoplasts that lack the U-box (Figure 1A and Figure 7A). In addition, patches were observed when masking the U-box in the full-length SAUL1 protein by N-terminally tagged GFP or by potential binding of E2 enzymes to the U-box [31] (Figure 7). However, when equipping GFP-SAUL1ARM_7–11_ with the N-terminal U-box, which allows for oligomerization, the U-box-GFP-SAUL1ARM_7–11_ fusion protein could not form patches; thus, oligomerization may prevent SAUL1 from tethering MVBs and/or the TP to the PM (Figure 7F).

Interestingly, GFP-SAUL1 and GFP-PUB43 did not form patches following expression in *Nicotiana benthamiana*. However, the tethering function of both proteins was induced by infection with the oomycete *P. capsici* [32]. It is tempting to speculate that the oomycete infection triggers a transition from SAUL1 tetramers, which exhibit a hidden non-functional tethering domain, to a differently organized conformation of SAUL1 that allows for tethering MVBs and/or the TP to the PM via its more accessible C-terminal ARM repeat domain. This transition could be a shift to elongated monomeric SAUL1 or an extended change in the conformation of tetramers. Generally, the association of vesicles with the PM has important functions in plant immunity, such as the secretion of molecules or release of exosomes. The fusion of secretory vesicles to the PM involves the exocyst complex [46]. The regulation of exocytosis likely involves the function of PUB-ARM proteins that target exocyst components [20,47]. It will be interesting to discover whether SAUL1-type ubiquitin ligases have a function in exocytosis. Additionally, the induction of the potential SAUL1 tethering function through oomycete infection may suggest that this PUB-ARM protein-mediated mechanism of vesicle association plays a role in the release of exosomes. The tethering of MVBs to the PM has been suggested as a potential source of extracellular vesicles in animals, fungi, and plants [48,49,50,51,52]. In plant-fungi interactions, exosomes appear to be important vehicles both organisms use to deliver molecules, such as small interfering RNAs and proteins, to each other [52,53,54]. In this article, we describe how the structural properties of SAUL1 may explain the tethering of MVB achieved upon oomycete infection. It is important to identify the changes in the oligomerization state, the conformation of SAUL1 in infected cells, and whether these changes require any modification of SAUL1, such as phosphorylation that regulates PUB22 dimerization [44]. The hypothesis that SAUL1 has a function in the release of exosomes for plant defense, specifically by mediating the association of MVBs with the PM, can be subject to future testing by analyzing the release of exosomes in *saul1-1 soc3* double mutants that do not express SAUL1 or show autoimmunity due to the lack of SOC3 activity [26].

## 4. Materials and Methods

### 4.1. Construction of Plasmids

Gateway Cloning^TM^ (Invitrogen, Carlsbad, CA, USA) generated the truncated C-terminal ARM domains, namely ARM7-10 and ARM8-11. The respective DNA fragments were amplified by PCR using Phusion^TM^ polymerase (Thermo Fisher Scientific, Waltham, MA, USA), and primers ARM7_At1g20780c + 1168f and ARM10_At1g20780c r + 1857r for ARM7–10, as well as ARM8_At1g20780c + 1297f, and ARM11_At1g20780c + 2241r for ARM8-11. The primer sequences are listed in Table S1 (Eurofins Genomics, Ebersberg, Germany). The PCR products were purified by NucleoSpin^®^ Gel and PCR Clean-up kits (Macherey–Nagel, Düren, Germany) and introduced into the pDONR221^TM^ backbone by BP^TM^ clonase-mediated gateway cloning. The obtained plasmids were sequenced to verify the expected sequence (GENEWIZ, Leipzig, Germany). The confirmed plasmids were used for LR clonase-mediated (Invitrogen, Carlsbad, CA, USA) gateway cloning to generate the respective expression constructs in the pMDC43 backbone, thus fusing the respective coding sequence of SAUL1 ARM domains to an N-terminal GFP-tag.

### 4.2. Site-Directed Mutagenesis

To exchange the three arginine residues at the amino acid positions 736, 737, and 775 in SAUL1, we applied site-directed mutagenesis in the pENTR^TM^ backbone carrying the *SAUL1* coding sequence. Complementary primer pairs (Table S1) were used with two mismatches for each arginine. A primer pair with four mismatches were used to introduce the respective double exchange. The Phusion^TM^ polymerase (Thermo Fisher Scientific, Waltham, MA, USA) introduced the mutation with 18 cycles for 4 min of denaturation at 98 °C followed by 1 min of annealing at 55 °C and an elongation of 12 min at 68 °C. To remove the template plasmid, a *DpnI* (Thermo Fisher Scientific, Waltham, MA, USA) digestion was performed for 30 min at 37 °C, and mutagenized plasmids were used for a heat shock transformation of *E. coli* Top10 cells. A successful introduction to the amino acid exchange was verified by sequencing (GENEWIZ, Leipzig, Germany). The confirmed plasmids were used for LR^TM^ clonase-mediated (Invitrogen, Carlsbad, CA, USA) gateway^TM^ cloning to generate the respective expression constructs in the pMDC43 backbone, thus fusing the SAUL1 coding sequences carrying the respective base exchanges for the mutation of the arginine residues to an N-terminal GFP tag. 

### 4.3. Protoplast Transformation

For the expression of GFP fusion proteins, the respective DNA constructs were transiently expressed in *A. thaliana* mesophyll protoplasts following the protocols described previously [26,31]. All fluorescent signals were analyzed by confocal laser scanning microscopy.

### 4.4. Confocal Laser Scanning Microscopy

Confocal laser scanning microscopy was applied using the Leica TCS SP8 Confocal Platform (Leica Microsystems, Wetzlar, Germany) to detect the fluorescence of green fluorescent protein (GFP) and yellow fluorescent protein (YFP) in living cells. For the excitation of GFP and YFP, laser light of 488 and 514 nm was used, respectively. The detection windows were 496 to 511 nm (GFP) and 521 to 535 nm (YFP), respectively. FM4-64 was excited at 488 nm and detected at 580 to 629 nm.

### 4.5. Expression and Purification of SAUL1

The coding sequence of SAUL1 was amplified by PCR from complementary DNA of *Arabidopsis thaliana* ecotype Col-0 and cloned to pGEX-6P-1 with BamHI/SalI (Table S1). *Escherichia coli* BL21 Star™ (DE3) were transformed with the pGEX-6P-1 construct to produce GST-tagged SAUL1 by recombinant gene expression. Liquid cultures in a 2YT medium containing ampicillin (100 µg mL^−1^) were incubated at 37 °C, and the expression was induced using a final concentration of 1 mM IPTG at an optical density at 600 nm of 1 AU. After 18 h, cells were harvested by centrifugation and resuspended in lysis buffer (50 mM Tris-HCl pH 9.0, 250 mM NaCl). Cells were lysed using lysozyme (1 mg mL^−1^) and sonication using a *Branson Sonifier ^®^ S250A* with an *S450A Cell Disruptor* (Emerson, St. Louis, MO, USA) with 50% output for five cycles with 30 s on and 30 s off. After pelleting cellular debris by centrifugation at 48000× *g* the supernatant was used for affinity chromatography using a GSTrap™ 4B 5 mL column and an *ÄKTA*™ *pure 25L* system (GE Healthcare, Chicago, IL, USA). GST-SAUL1 was eluted using 50 mM Tris-HCl pH 9.0, 250 mM NaCl, 50 mM glutathione, and the tag was removed using *PreScission*™ protease (GE Healthcare, Chicago, IL, USA). Afterward, SAUL1 was purified by size-exclusion chromatography using a *HiLoad*™ *16/600 200pg* column (GE Healthcare, Chicago, IL, USA). In a final step, SAUL1 was concentrated to the desired protein concentration by centrifugal concentration.

### 4.6. Dynamic Light Scattering (DLS)

To analyze solution dispersity and oligomeric states of SAUL1, DLS experiments were conducted using the SpectroSize™ 300 (Xtal Concepts, Hamburg, Germany). Protein solutions were measured using a Hellma™ Far UV quartz cuvette with a light path length of 10 mm. Scattering was measured 20 times for 20 s at 20 °C using a 660 nm diode laser. The time-dependent fluctuations of the scattering intensity at an angle of 90° were autocorrelated and evaluated using the CONTIN algorithm [55]. The average hydrodynamic radii distributions were calculated using the Stokes–Einstein Equation.

### 4.7. Circular Dichroism (CD) Spectroscopy

To obtain insights into the secondary structure of SAUL1, CD spectroscopy measurements were performed using the *J-815* spectrometer (JACO International, Tokyo, Japan). Protein solutions were measured in 1:25 diluted SEC buffer containing 50 mM Tris-HCl pH 9.0, 250 mM NaCl, and 0.1 mM AEBSF. The instrument was calibrated according to the manufacturer’s specifications. The ellipticity of the sample was measured in a 1 mm quartz cuvette in a wavelength interval ranging from 185 to 260 nm. For each sample, 15 spectra were averaged. The baseline recorded for the corresponding buffer was subtracted. The mean residue ellipticity ([θ]) was calculated as [θ] = (m° × *M*_W_/number of amino acids)/(10 × l × c) where m° is the ellipticity in millidegrees, *M*_W_ is the molecular weight in Da, l is the light path length in cm and c is the protein concentration in mg mL^−1^. The analysis was performed as described previously [56].

### 4.8. Small-Angle X-ray Scattering (SAXS) and Inline SEC-SAXS

SAXS data were collected at the synchrotron beamline P12 operated by EMBL Hamburg at the PETRA III storage ring (DESY, Hamburg, Germany). Scattering data was collecting using a photon-counting *Pilatus3 X 2M* pixel detector (Dectris, Baden-Daettwil, Switzerland) with a sample-detector distance of 3.1 m. A scattering vector *q* (where *q* = 4π sin *θ*/*λ*, 2*θ* is the scattering angle and *λ* is the radiation wavelength) ranging from 0.03 to 4.8 nm was recorded (Table S2).

For SAXS batch measurements, SAUL1 solutions in the form of a concentration series between 1 and 10 mg mL^−1^ were used. The scattering of protein solutions and corresponding buffers was recorded successively 20 times for 45 ms. As the first data processing step, all 20 measurements were normalized to H_2_O and averaged. Prior to the measurements, different concentrations of bovine serum albumin (BSA) were measured to verify beamline operation and data processing. In addition, forward scattering intensities were used to calculate theoretical *M*_W_-values for the proteins of interest. The theoretical *M*_W_ is defined as *M_WI_* = *M_WS_*/*I*(0)*_S_* × *I*(0)*_I_* where *M_WS_* is the molecular weight of BSA in Da; *I*(0)*_S_* is the averaged forward scattering intensity of BSA; and *I*(0)*_I_* is the forwards scattering intensity of the protein of interest [57]. 

The SEC-SAXS measurement was performed using a *Superose*™ *6 Increase 10/300 GL* (GE Healthcare, Chicago, IL, USA) column with a flow rate of 0.5 mL min^−1^. A continuous 1 s data frame measurement of 50 µl of a sample with 120 frames mL^−1^ was performed (Table S3). Data were preprocessed using *CHROMIXS* [58]. Twenty-five frames from each sample were selected manually. Fifty corresponding buffer frames were selected automatically. The buffer scattering data was subtracted for both measurements. All these steps were performed using the *SASFLOW* pipeline.

### 4.9. Data Processing

All further evaluation steps were performed using the *ATSAS* suite [59,60]. As the next step, the radius of gyration (*R_g_*) and the forward scattering intensity (*I_0_*) were calculated using the Guinier fit by applying *AUTORG*. Using an indirect Fourier transformation, the P(*r*) function was calculated by *DATGNOM*. The maximum diameter (*d_max_*) was defined manually. These programs were used as part of the *PRIMUSQT* package. Flory’s equation was used to calculate theoretical radii of gyration (*R_g_*) for proteins, which is defined as *R_g_* = *R_0_* × *N^v^* where *R_0_* is a constant depending on the type of protein, in Å, *N* is the number of amino acid residues, and *v* is a scaling factor [61]. To evaluate the fit, standardized residuals (*Δ/σ*) were plotted as *∆/σ*= (*I_obs_* (*s*) − *I_exp_* (*s*))/(*σ*(*I_exp_* (*s*))) where *I_obs_*(*s*) is the detected scattering intensity; *I_exp_*(*s*) is the expected intensity, and *σ*(*I_exp_*(*s*)) is the standard deviation of the expected intensities. 

In the next step, the calculated radius distribution was used to generate ab initio models of the protein investigated using *GASBOR*. Ab initio models were generated 20 times and compared using *DAMAVER*. The final model was chosen based on the normalized spatial discrepancy. The full-length sequence of SAUL1 was subjected to homology-based modeling using I-TASSER [62]. Threading templates with up to 22% local sequence identity were identified in the protein database using default modeling parameters. The confidence of the best model is characterized by a C-score of −1.34, an estimated TM-score of 0.55 ± 0.15, and an estimated RMSD of 11.6 ± 4.5 Å. Different programs were applied to adjust *I-TASSER*-derived in silico models to the scattering data. In the case of batch measurements, *SASREF* was used to refine the models and obtain different oligomeric structures. The composition was determined using *OLIGOMER* and evaluated with CRYSOL. In case of SEC-SAXS measurements, EOM was used to account for flexible regions in the protein. Therefore, the protein was divided into seven domains, predicted to be more ordered following the *I-TASSER* modeling. These domains contained amino acids 20 to 54, 65 to 126, 142 to 211, 226 to 382, 390 to 427, 434 to 698, and 706 to 784. The inter-domain regions were defined as flexible and allowed for adjusting the model to the obtained scattering data.

## Supplementary Materials

The following are available online https://www.mdpi.com/article/10.3390/ijms22179455/s1, Table S1: Primers; Table S2: SAXS data collection parameters; Table S3: SEC-SAXS data collection parameters; Figure S1: Expression, isolation, and purification of recombinant SAUL1; Figure S2: CD spectroscopy analysis of SAUL1 and SAUL1 R736A; Figure S3: *EOM* analysis of SAUL1 in SEC-SAXS measurements; Figure S4: Plasma membrane localization of SAUL1 R736A/R737A/R775A; Figure S5: Scattering data of SAUL1 in a batch experiment; Figure S6: Oligomeric analysis of SAUL1; Figure S7: In silico structure of PUB43 modeled on SEC-SAXS data of SAUL1 and comparison to the SAUL1 model.

## Abbreviations

PUB-ARM	Plant U-box armadillo repeat
Ub	Ubiquitin
SAUL1	SENESCENCE ASSOCIATED UBIQUITIN E3 LIGASE1
MVBs	Multi-vesicular bodies
SAXS	Small-angle X-ray scattering
PRR	Pattern recognition receptors
PAMP	Pathogen-associated molecular pattern
PTI	PAMP-triggered immunity
ETI	Effector-triggered immunity
SOC3	Suppressors of chs1-2, 3
PM	Plasma membrane
TP	Tonoplast
SEC	Size exclusion chromatography
DLS	Dynamic light scattering
EOM	Ensemble optimization method
ARM	Armadillo
UBC	Ubiquitin-conjugating

## References

[1] Jones J.D., Dangl J.L. (2006). The plant immune system. Nature.

[2] Boller T., Felix G. (2009). A renaissance of elicitors: Perception of microbe-associated molecular patterns and danger signals by pattern-recognition receptors. Annu. Rev. Plant Biol..

[3] Monaghan J., Zipfel C. (2012). Plant pattern recognition receptor complexes at the plasma membrane. Curr. Opin. Plant Biol..

[4] Galan J.E., Lara-Tejero M., Marlovits T.C., Wagner S. (2014). Bacterial type III secretion systems: Specialized nanomachines for protein delivery into target cells. Annu. Rev. Microbiol..

[5] Macho A.P., Zipfel C. (2015). Targeting of plant pattern recognition receptor-triggered immunity by bacterial type-III secretion system effectors. Curr. Opin. Microbiol..

[6] Li X., Kapos P., Zhang Y. (2015). NLRs in plants. Curr. Opin. Immunol..

[7] Azevedo C., Santos-Rosa M.J., Shirasu K. (2001). The U-box protein family in plants. Trends Plant Sci..

[8] Zhou B., Zeng L. (2017). Conventional and unconventional ubiquitination in plant immunity. Mol. Plant Pathol..

[9] Trujillo M. (2018). News from the PUB: Plant U-box type E3 ubiquitin ligases. J. Exp. Bot..

[10] Hatakeyama S., Yada M., Matsumoto M., Ishida N., Nakayama K.I. (2001). U box proteins as a new family of ubiquitin-protein ligases. J. Biol. Chem..

[11] Pruneda J.N., Littlefield P.J., Soss S.E., Nordquist K.A., Chazin W.J., Brzovic P.S., Klevit R.E. (2012). Structure of an E3:E2~Ub complex reveals an allosteric mechanism shared among RING/U-box ligases. Mol. Cell.

[12] Huber A.H., Nelson W.J., Weis W.I. (1997). Three-dimensional structure of the armadillo repeat region of beta-catenin. Cell.

[13] Coates J.C. (2003). Armadillo repeat proteins: Beyond the animal kingdom. Trends Cell Biol..

[14] Lu D., Lin W., Gao X., Wu S., Cheng C., Avila J., Heese A., Devarenne T.P., He P., Shan L. (2011). Direct ubiquitination of pattern recognition receptor FLS2 attenuates plant innate immunity. Science.

[15] Liao D., Cao Y., Sun X., Espinoza C., Nguyen C.T., Liang Y., Stacey G. (2017). Arabidopsis E3 ubiquitin ligase PLANT U-BOX13 (PUB13) regulates chitin receptor LYSIN MOTIF RECEPTOR KINASE5 (LYK5) protein abundance. New Phytol..

[16] Kong L., Cheng J., Zhu Y., Ding Y., Meng J., Chen Z., Xie Q., Guo Y., Li J., Yang S. (2015). Degradation of the ABA co-receptor ABI1 by PUB12/13 U-box E3 ligases. Nat. Commun..

[17] Liu J., Li W., Ning Y., Shirsekar G., Cai Y., Wang X., Dai L., Wang Z., Liu W., Wang G.-L. (2012). The U-Box E3 ligase SPL11/PUB13 is a convergence point of defense and flowering signaling in plants. Plant Physiol..

[18] Wang J., Grubb L.E., Wang J., Liang X., Li L., Gao C., Ma M., Feng F., Li M., Li L. (2018). A regulatory module controlling homeostasis of a plant immune kinase. Mol. Cell.

[19] Trujillo M., Ichimura K., Casais C., Shirasu K. (2008). Negative regulation of PAMP-triggered immunity by an E3 ubiquitin ligase triplet in Arabidopsis. Curr. Biol..

[20] Stegmann M., Anderson R.G., Ichimura K., Pecenkova T., Reuter P., Zarsky V., McDowell J.M., Shirasu K., Trujillo M. (2012). The ubiquitin ligase PUB22 targets a subunit of the exocyst complex required for PAMP-triggered responses in Arabidopsis. Plant Cell.

[21] Gonzalez-Lamothe R., Tsitsigiannis D.I., Ludwig A.A., Panicot M., Shirasu K., Jones J.D. (2006). The U-box protein CMPG1 is required for efficient activation of defense mechanisms triggered by multiple resistance genes in tobacco and tomato. Plant Cell.

[22] Yang C.-W., Gonzalez-Lamothe R., Ewan R.A., Rowland O., Yoshioka H., Shenton M., Ye H., O’Donnell E., Jones J.D., Sadanandom A. (2006). The E3 ubiquitin ligase activity of arabidopsis PLANT U-BOX17 and its functional tobacco homolog ACRE276 are required for cell death and defense. Plant Cell.

[23] Ishikawa K., Yamaguchi K., Sakamoto K., Yoshimura S., Inoue K., Tsuge S., Kojima C., Kawasaki T. (2014). Bacterial effector modulation of host E3 ligase activity suppresses PAMP-triggered immunity in rice. Nat. Commun..

[24] Wang J., Qu B., Dou S., Li L., Yin D., Pang Z., Zhou Z., Tian M., Liu G., Xie Q. (2015). The E3 ligase OsPUB15 interacts with the receptor-like kinase PID2 and regulates plant cell death and innate immunity. BMC Plant Biol..

[25] Han P.-L., Dong Y.-H., Gu K.-D., Yu J.-Q., Hu D.-G., Hao Y.-J. (2019). The apple U-box E3 ubiquitin ligase MdPUB29 contributes to activate plant immune response to the fungal pathogen Botryosphaeria dothidea. Planta.

[26] Tong M., Kotur T., Liang W., Vogelmann K., Kleine T., Leister D., Brieske C., Yang S., Lüdke D., Wiermer M. (2017). E3 ligase SAUL1 serves as a positive regulator of PAMP-triggered immunity and its homeostasis is monitored by immune receptor SOC3. New Phytol..

[27] Raab S., Drechsel G., Zarepour M., Hartung W., Koshiba T., Bittner F., Hoth S. (2009). Identification of a novel E3 ubiquitin ligase that is required for suppression of premature senescence in Arabidopsis. Plant J..

[28] Disch E.M., Tong M., Kotur T., Koch G., Wolf C.A., Li X., Hoth S. (2016). Membrane-associated ubiquitin ligase SAUL1 suppresses temperature- and humidity-dependent autoimmunity in Arabidopsis. Mol. Plant Microbe Interact..

[29] Vogelmann K., Subert C., Danzberger N., Drechsel G., Bergler J., Kotur T., Burmester T., Hoth S. (2014). Plasma membrane-association of SAUL1-type plant U-box armadillo repeat proteins is conserved in land plants. Front. Plant Sci..

[30] Liang W., van Wersch S., Tong M., Li X. (2019). TIR-NB-LRR immune receptor SOC3 pairs with truncated TIR-NB protein CHS1 or TN2 to monitor the homeostasis of E3 ligase SAUL1. New Phytol..

[31] Drechsel G., Bergler J., Wippel K., Sauer N., Vogelmann K., Hoth S. (2011). C-terminal armadillo repeats are essential and sufficient for association of the plant U-box armadillo E3 ubiquitin ligase SAUL1 with the plasma membrane. J. Exp. Bot..

[32] Tao K., Waletich J.R., Wise H., Arredondo F., Tyler B.M. (2019). Tethering of multi-vesicular bodies and the tonoplast to the plasma membrane in plants. Front. Plant Sci..

[33] Andersen P., Kragelund B.B., Olsen A.N., Larsen F.H., Chua N.H., Poulsen F.M., Skriver K. (2004). Structure and biochemical function of a prototypical Arabidopsis U-box domain. J. Biol. Chem..

[34] Zhang M., Windheim M., Roe S.M., Peggie M., Cohen P., Prodromou C., Pearl L.H. (2005). Chaperoned ubiquitylation--crystal structures of the CHIP U box E3 ubiquitin ligase and a CHIP-Ubc13-Uev1a complex. Mol. Cell.

[35] Kooi C.W.V., Ohi M.D., Rosenberg J.A., Oldham M.L., Newcomer M.E., Gould K.L., Chazin W.J. (2006). The Prp19 U-box crystal structure suggests a common dimeric architecture for a class of oligomeric E3 ubiquitin ligases. Biochemistry.

[36] Tu D., Li W., Ye Y., Brunger A.T. (2007). Structure and function of the yeast U-box-containing ubiquitin ligase Ufd2p. Proc. Natl. Acad. Sci. USA.

[37] Graewert M.A., Franke D., Jeffries C.M., Blanchet C.E., Ruskule D., Kuhle K., Flieger A., Schafer B., Tartsch B., Meijers R. (2015). Automated pipeline for purification, biophysical and x-ray analysis of biomacromolecular solutions. Sci. Rep..

[38] Mudgil Y., Shiu S.H., Stone S.L., Salt J.N., Goring D.R. (2004). A large complement of the predicted Arabidopsis ARM repeat proteins are members of the U-box E3 ubiquitin ligase family. Plant Physiol..

[39] Tewari R., Bailes E., Bunting K.A., Coates J.C. (2010). Armadillo-repeat protein functions: Questions for little creatures. Trends Cell Biol..

[40] Ritco-Vonsovici M., Ababou A., Horton M. (2007). Molecular plasticity of beta-catenin: New insights from single-molecule measurements and MD simulation. Protein Sci..

[41] Zhang Z., Lin K., Gao L., Chen L., Shi X., Wu G. (2011). Crystal structure of the armadillo repeat domain of adenomatous polyposis coli which reveals its inherent flexibility. Biochem. Biophys. Res. Commun..

[42] Antignani V., Klocko A.L., Bak G., Chandrasekaran S.D., Dunivin T., Nielsen E. (2015). Recruitment of PLANT U-BOX13 and the PI4Kbeta1/beta2 phosphatidylinositol-4 kinases by the small GTPase RabA4B plays important roles during salicylic acid-mediated plant defense signaling in Arabidopsis. Plant Cell.

[43] Yee D., Goring D.R. (2009). The diversity of plant U-box E3 ubiquitin ligases: From upstream activators to downstream target substrates. J. Exp. Bot..

[44] Furlan G., Nakagami H., Eschen-Lippold L., Jiang X., Majovsky P., Kowarschik K., Hoehenwarter W., Lee J., Trujillo M. (2017). Changes in PUB22 ubiquitination modes triggered by MITOGEN-ACTIVATED PROTEIN KINASE3 dampen the immune response. Plant Cell.

[45] Jung C., Zhao P., Seo J.S., Mitsuda N., Deng S., Chua N.H. (2015). PLANT U-BOX PROTEIN10 regulates MYC2 stability in Arabidopsis. Plant Cell.

[46] Zarsky V., Kulich I., Fendrych M., Pecenkova T. (2013). Exocyst complexes multiple functions in plant cells secretory pathways. Curr. Opin. Plant Biol..

[47] Seo D.H., Ahn M.Y., Park K.Y., Kim E.Y., Kim W.T. (2016). The N-Terminal UND Motif of the Arabidopsis U-Box E3 Ligase PUB18 is critical for the negative regulation of ABA-mediated stomatal movement and determines its ubiquitination specificity for exocyst subunit Exo70B1. Plant Cell.

[48] An Q., van Bel A.J., Hückelhoven R. (2007). Do plant cells secrete exosomes derived from multivesicular bodies?. Plant Signal. Behav..

[49] Hanson P.I., Cashikar A. (2012). Multivesicular body morphogenesis. Annu. Rev. Cell Dev. Biol..

[50] Colombo M., Raposo G., Thery C. (2014). Biogenesis, secretion, and intercellular interactions of exosomes and other extracellular vesicles. Annu. Rev. Cell Dev. Biol..

[51] Samuel M., Bleackley M., Anderson M., Mathivanan S. (2015). Extracellular vesicles including exosomes in cross kingdom regulation: A viewpoint from plant-fungal interactions. Front. Plant Sci..

[52] Rutter B.D., Innes R.W. (2018). Extracellular vesicles as key mediators of plant-microbe interactions. Curr. Opin. Plant Biol..

[53] Cai Q., Qiao L., Wang M., He B., Lin F.M., Palmquist J., Huang S.-D., Jin H. (2018). Plants send small RNAs in extracellular vesicles to fungal pathogen to silence virulence genes. Science.

[54] Baldrich P., Rutter B.D., Karimi H.Z., Podicheti R., Meyers B.C., Innes R.W. (2019). Plant extracellular vesicles contain diverse small RNA species and are enriched in 10- to 17-Nucleotide “Tiny” RNAs. Plant Cell.

[55] Provencher S.W. (1982). Contin: A general purpose constrained regularization program for inverting noisy linear algebraic and itegral equations. Comput. Phys. Commun..

[56] Reed J., Reed T.A. (1997). A set of constructed type spectra for the practical estimation of peptide secondary structure from circular dichroism. Anal. Biochem..

[57] Mylonas E., Svergun D.I. (2007). Accuracy of molecular mass determination of proteins in solution by small-angle X-ray scatterig. J. Appl. Crsystallogr..

[58] Panjkovich A., Svergun D.I. (2016). Deciphering conformational transitions of proteins by small angle X-ray scattering and normal mode analysis. Phys. Chem. Chem. Phys..

[59] Petoukhov M.V., Konarev P.V., Kikhney A.G., Svergun D.I. (2007). ATSAS 2.1—Towards automated and web-supported small-angle scattering data analysis. J. Appl. Crystallogr..

[60] Franke D., Petoukhov M.V., Konarev P.V., Panjkovich A., Tuukkanen A., Mertens H.D.T., Kikhney A.G., Hajizadeh N.R., Franklin J.M., Jeffries C.M. (2017). ATSAS 2.8: A comprehensive data analysis suite for small-angle scattering from macromolecular solutions. J. Appl. Crystallogr..

[61] Flory P.J. (1953). Principles of Polymer Chemistry.

[62] Yang J., Zhang Y. (2015). I-TASSER server: New development for protein structure and function predictions. Nucleic Acids Res..

