# Supplementary material for: Structural Features of a Full-Length Ubiquitin Ligase Responsible for the Formation of Patches at the Plasma Membrane
Source: Int J Mol Sci. 2021 Aug 31;22(17):9455. doi: 10.3390/ijms22179455 (PMC8431560; doi:10.3390/ijms22179455)
Supplement: Supplementary file 1 [file ijms-22-09455-s001.zip › ijms-1345017-supplementary.pdf]

Structural features of a full-length ubiquitin ligase responsible for the formation of patches at the plasma membrane

Jan Knop[1], Tim Lienemann[1], Haifa El-Kilani[2, 3], Sven Falke[2, 3], Catharina Krings[1], Maria Sindalovskaya[1], Johannes Bergler[1], Christian Betzel[2, 3], Stefan Hoth[1, *]

[1] Molecular Plant Physiology, Institute of Plant Science and Microbiology, Universität Hamburg, 22609 Hamburg, Germany

[2] Institute of Biochemistry and Molecular Biology, Universität Hamburg, 20146 Hamburg, Germany

[3] Laboratory for Structural Biology of Infection and Inflammation, c/o DESY, 22607 Hamburg, Germany

[*] Correspondence: Stefan Hoth (stefan.hoth@uni-hamburg.de)

List of material

Supplementary Table 1. Primers
Supplementary Table 2. SAXS data collection parameters
Supplementary Table 3. SEC-SAXS data collection parameters
Supplementary Figure 1. Expression, isolation and purification of recombinant SAUL1.
Supplementary Figure 2. CD spectroscopy analysis of SAUL1 and SAUL1 R736A.
Supplementary Figure 3. *EOM* analysis of SAUL1 in SEC-SAXS measurements.
Supplementary Figure 4. Plasma membrane localization of SAUL1 R736A/R737A/R775A.
Supplementary Figure 5. Scattering data of SAUL1 in a batch experiment.
Supplementary Figure 6. Oligomeric analysis of SAUL1.
Supplementary Figure 7. *In silico* structure of PUB43 modelled on SEC-SAXS data of SAUL1 and comparison to the SAUL1 *in silico* model.

Supplementary materials

Supplementary Table 1

Primers

ID	Sequence (5' to 3'), restriction sites and sequences for recombination are marked in red
ARM7_At1g20780c+1168f	GGGGACAAGTTTGTACAAAAAAGCAGGCTATATGGACTTTGACAAAGCCACTCTTG
ARM10_At1g20780c r+1857r	GGGGACCACTTTGTACAAGAAAGCTGGGTATCAAAGTGAAAGATTCTCTAAAGCC
ARM8_At1g20780c +1297f	GGGGACAAGTTTGTACAAAAAAGCAGGCTATATGACAGTTCCAAAAGTTGTTTATGC
ARM11_At1g20780c +2241r	GGGGACCACTTTGTACAAGAAAGCTGGGTATCAAAGCAAGATCCTTTCCACCATC
SDM_ At1g20780c R736A fw	GAGGCTGACAGCGAGAGCGGTTTGGATGGTG
SDM_ At1g20780c R736A rev	CACCATCCAAACCGCTCTCGCTGTCAGCCT
SDM_ At1g20780c R737A fw	GGCTGACAAGGGCAGCGGTTTGGATGG
SDM_ At1g20780c R737A rev	CCATCCAAACCGCTGCCCTTGTCAGCC
SDM_ At1g20780c R775A fw	CGCTGATTTTGCAACGAGACAGATCGCTGAG
SDM_ At1g20780c R775A rev	CTCAGCGATCTGTCTCGTTGCAAAATCAGCG
SDM_ At1g20780c R736+737A) fw	GGCTGACAGCGGCAGCGGTTTGGATGG
SDM_ At1g20780c R736+737A rev	CCATCCAAACCGCTGCCCTTGTCAGCC
At1g20780c+1fBamHI	GCGGATCCATGGTTGGAAGCTCGGATGG
At1g20780c+2403rSalI	GCGTCGACCTATGCGATGTTTGGG

Supplementary Table 2

SAXS data collection parameters

X-ray source	PETRA III (DESY, Hamburg, Germany), Beamline P12
Detector	Photon counting *Pilatus3 X 2M* pixel detector (253 x 288 mm^2) (Dectris, Baden-Daettwil, Switzerland)
Sample-detector-distance (m)	3.1
Wavelength (nm)	0.124
Focal spot (mm)	0.2 × 0.12
s range (nm^{-1})	0.03-4.8
s-axis calibration	Silver behenate
Exposure time (ms)	45
Sample temperature (°C)	20

Supplementary Table 3

SEC-SAXS data collection parameters

HPLC	*1260 Infinity II Bio-Inert* sytem (Agilent, Santa Clara, United States)
SEC column	*Superose™ 6 Increase 10/300 GL* (GE Healthcare, Chicago, United States)
Injection volume (µl)	50
Flow rate (ml min^{-1})	0.5
Frame rate (frames ml^{-1})	120
Exposure time	Continuous1 s data-frame measurements

Supplementary Figure 1

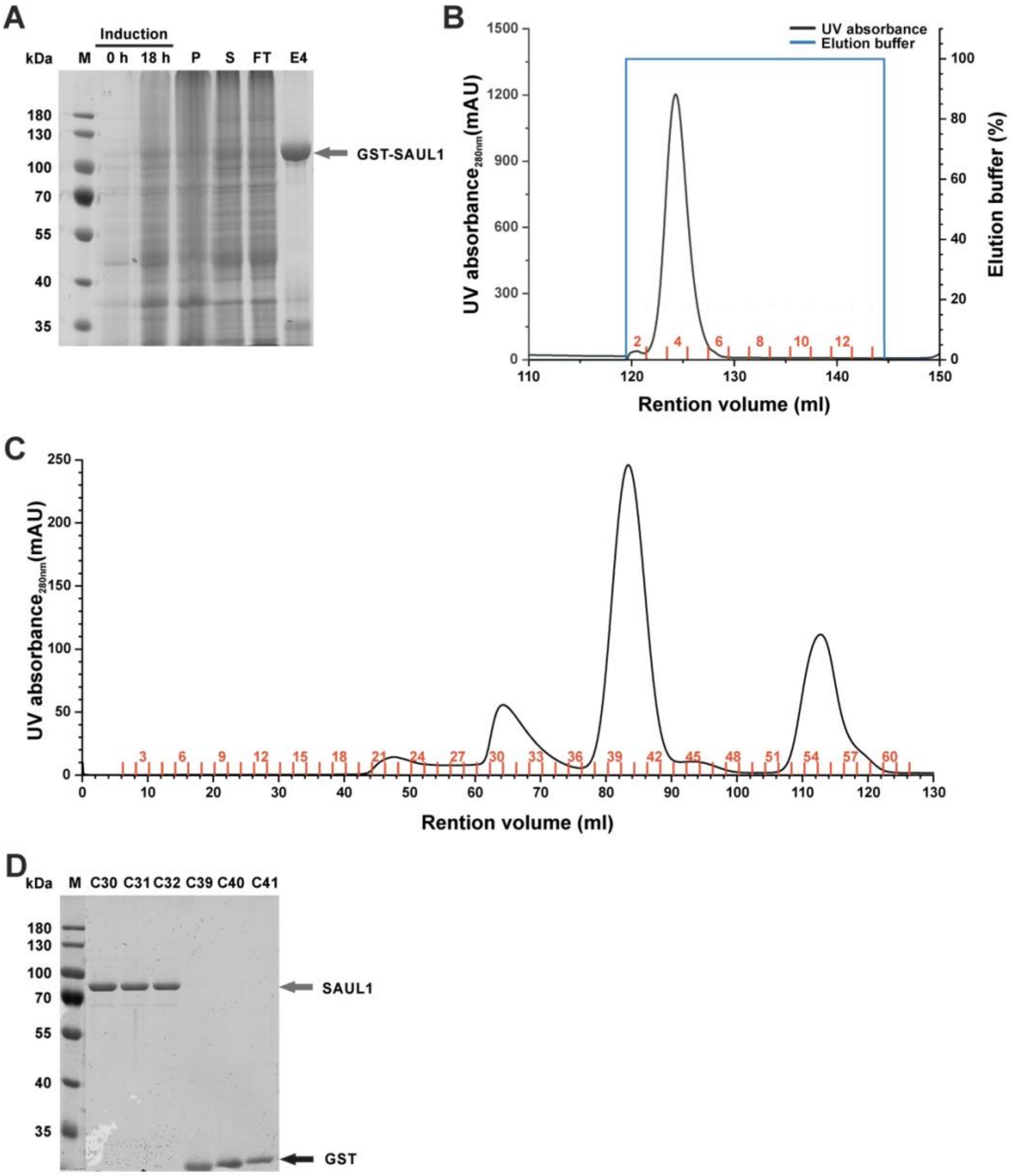

Supplementary Figure 1. Expression, isolation and purification of recombinant SAUL1. (A) SDS-PAGE analysis of recombinant SAUL1 purification. The lanes containing the marker M, the pellet P, the supernatant S, and the flow-through FT are indicated. Elution (E4) was performed using the SAUL1 buffer containing 50 mM glutathione. GST-tag removal using the *PreScission™* (GE Healthcare, Chicago, United States). (B) Affinity chromatography of GST-SAUL1 using the *ÄKTA™ pure 25L* (GE Healthcare, Chicago, United States) system and a GSTrap™ 4B 5 ml (GE Healthcare, Chicago, United States) column. Bound recombinant proteins were eluted using 50 mM glutathione (blue curve). (C) Separation of SAUL1 and the GST-tag, which were cleaved with *PreScission™*, using size-exclusion chromatography. Size exclusion chromatography of digested SAUL1 using a *HiLoad™ 16/600 200pg* (GE Healthcare, Chicago, United States) column and a sample volume of 5 ml. Peaks are detectable in fractions 21-23 (GST-SAUL1), 30-34 (SAUL1), 38-43 (GST-tag), 44-46 (nothing detectable by SDS-PAGE) and 53-58 (nothing detectable by SDS-PAGE). (D) SDS-PAGE of the size-exclusion chromatography fractions of the first (E30-33) and the second peak (E39-41). GST-SAUL1 has a theoretical size of 115.2 kDa (dark grey arrow), SAUL1 of 88.7 kDa (black arrow), and GST of 26.5 kDa (light grey arrow).

Supplementary Figure 2

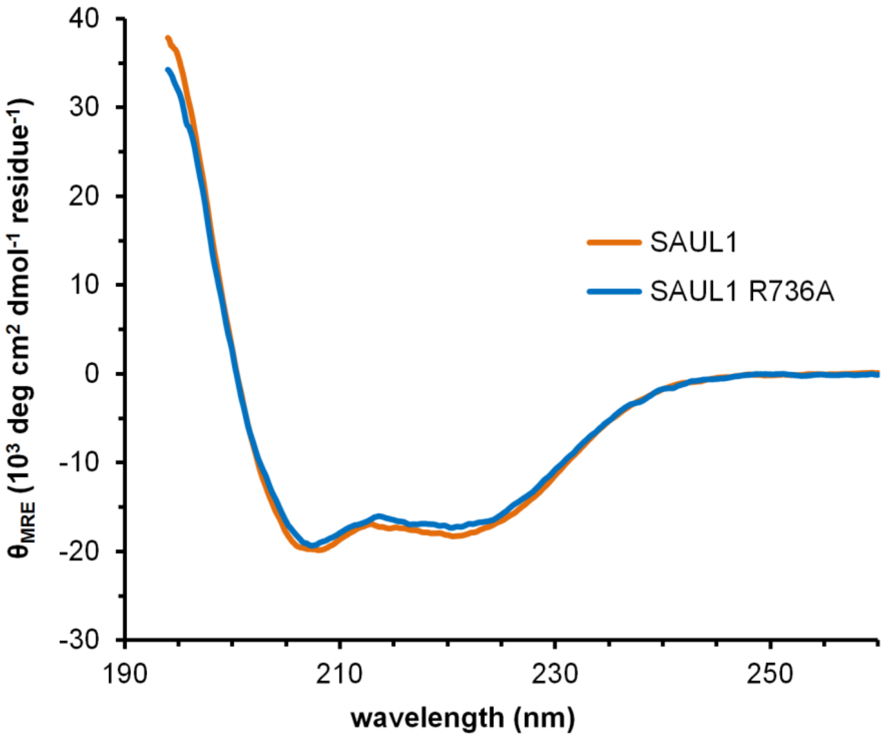

Supplementary Figure 2. CD spectroscopy analysis of SAUL1 and SAUL1 R736A.
CD spectra of SAUL1 (blue) and SAUL1 R736A (orange) were depicted as mean residue ellipticity (θ_{MRE}).

Supplementary Figure 3

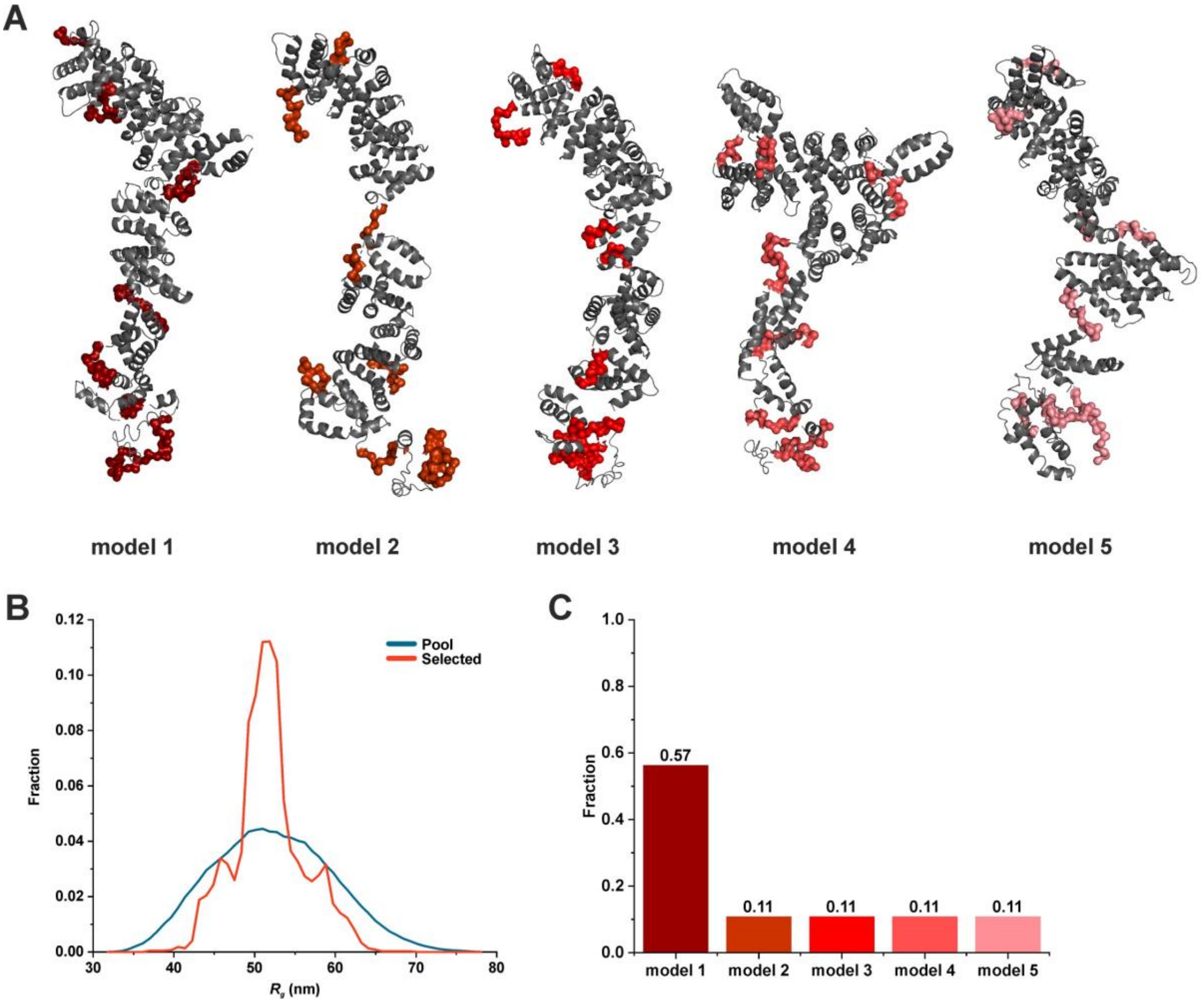

Supplementary Figure 3. ***EOM* analysis of SAUL1 in SEC-SAXS measurements.**
(A) Five different SAUL1 models were obtained by fitting an *in silico I-TASSER* model to the experimental SEC-SAXS data using *EOM*. (B) Fraction analysis of the R_g values of these models, which were generated at the start of the *EOM* calculation (Pool), and those fitted best to the experimental data (Selected). (C) Fraction analysis of all five models.

Supplementary Figure 4

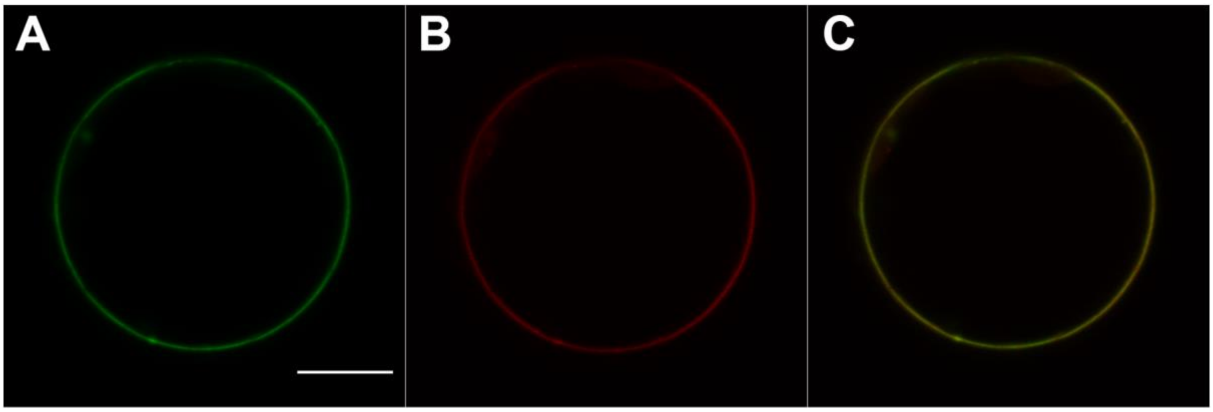

Supplementary Figure 4. Plasma membrane localization of SAUL1 R736A/R737A/R775A. (A) GFP fluorescence of GFP-SAUL1 R736A/R737A/R775A following expression in mesophyll protoplasts. (B) Fluorescence of FM4-64 in protoplasts expressing GFP-SAUL1 R736A/R737A/R775A (C). Merged picture of the pictures from (A) and (B). Scale bar represents 10 µm.

Supplementary Figure 5

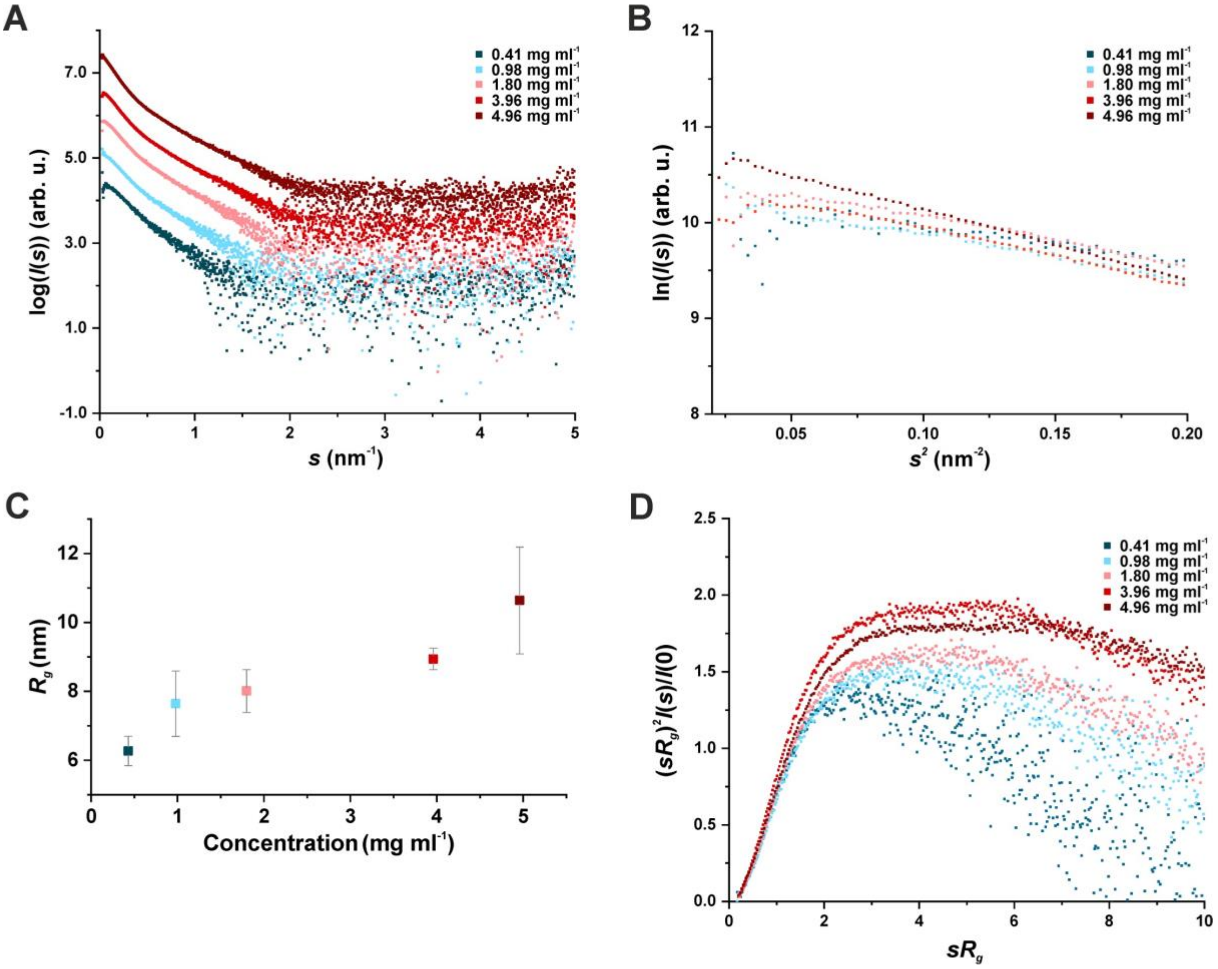

Supplementary Figure 5. Scattering data of SAUL1 in a batch experiment.
(A) Two-dimensional Log-linear plot of *I*(*s*) versus *s* of the scattering data of different concentrations. Curves were plotted with an offset by being multiplied by a factor of 5 compared to the previous scattering curve. (B) Guinier plot of the SAXS data around *I*(0). (C) Plot of the different Guinier-derived R_g values and standard deviations against their corresponding concentrations. (D) Dimensionless Kratky plot with the intensities normalized to the forward scattering intensity (*I*(0)) and R_g.

Supplementary Figure 6

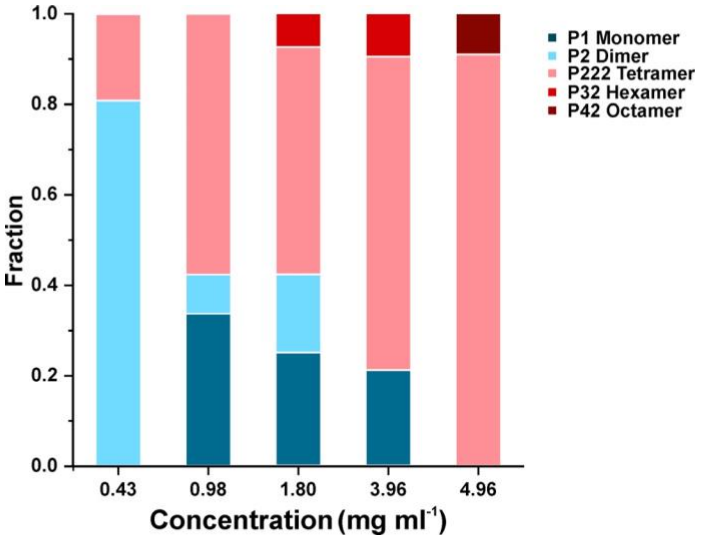

Supplementary Figure 6. Oligomeric analysis of SAUL1.
Analysis of polydisperse SAXS data of SAUL1 using OLIGOMER. Fractions of different oligomeric states of SAUL1 at various protein concentrations are shown.

Supplementary Figure 7

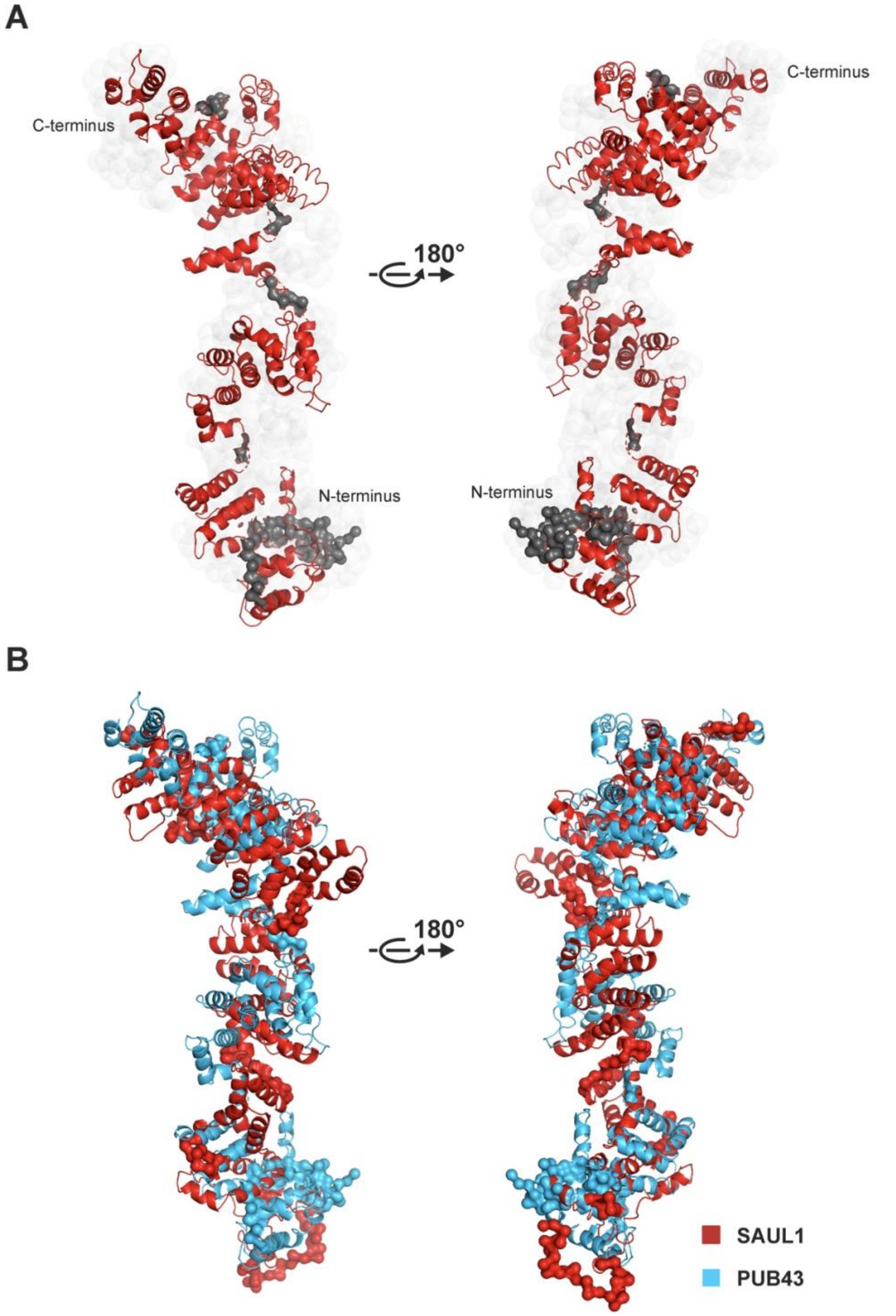

Supplementary Figure 7. *In silico* structure of PUB43 modelled on SEC-SAXS data of SAUL1 and comparison to the SAUL1 *in silico* model.
(A) *I-TASSER* derived *in silico* model of PUB43, which was subdivided into different domains upon an *InterPro* analysis and refined using *EOM*. Depicted are α-helices (red), β-sheets (blue), loops (grey) and flexible regions (grey). (B) Alignment of the *I-TASSER* derived *in silico* models of SAUL1 (red) and PUB43 (blue).
